# De-alloying Behavior of Mg–Al alloy in Sulphuric Acid and Acetic Acid Aqueous Solutions

**DOI:** 10.3390/ma12132046

**Published:** 2019-06-26

**Authors:** Yonggang Li, Yinghui Wei, Shengqiang Yang

**Affiliations:** 1 College of Mechanical and Vehicle Engineering, Taiyuan University of Technology, Taiyuan 030024, China; 2 Taiyuan Heavy Machinery Group Co., Ltd, Taiyuan 030024, China; 3 College of Materials Science and Engineering, Taiyuan University of Science and Technology, Taiyuan 030024, China

**Keywords:** acid solutions, SEM, acid corrosion, de-alloying

## Abstract

The fabricated Mg–Al alloy consists of α-Mg phase and Mg–Mg_17_Al_12_ eutectic phase. The corrosion behavior of cast Mg–Al alloy in sulphuric acid (H_2_SO_4_) and acetic acid (HAc) aqueous solutions was investigated. The Mg–Al alloy shows general corrosion in H_2_SO_4_ solution, and the α-Mg dendrites revealed a slightly faster corrosion rate than that of the eutectics. In HAc solution, the alloy shows an obvious selective corrosion characteristic, with the α-Mg dendrites being corroded preferentially. Grain orientation plays an important role in corrosion behavior of the alloy in the HAc solutions.

## 1. Introduction

Magnesium and its alloys have attracted huge interest as engineering materials for industry and electronics applications because of their low density and good mechanical properties [1,2,3]. The poor corrosion resistance has limited the wide use of magnesium alloys [4,5,6]. Reducing the impurities is an effective way for improving the corrosion resistance of these alloys [7,8,9,10,11]. The corrosion behavior of Mg alloys is highly related to their microstructures. Intermetallic phases, formed during casting, affect the corrosion process [12].

Generally speaking, the second phases in Mg alloys have been investigated by the following two main methods. On the one hand, researchers have prepared many alloys with different second-phase contents and distributions through various fabrication processes and element content control methods. Studying the corrosion resistance of these alloys can aid in understanding the roles of second phases in the corrosion process [13,14,15]. On the other hand, single-phase alloys are prepared with the same chemical compositions as the second phases of interest. By investigating the corrosion behavior of these single-phase alloys, the corrosion mechanisms of Mg alloys can be understood deeply [12,16].

Among the various commercially available alloys, the Mg–Al–Mn and Mg–Al–Zn series are widely used as engineering material [17,18,19]. The main alloying element in both alloy series is Al. The distribution of the Al can affect the corrosion behavior of Mg alloys. Al exists in Mg alloys in two ways: As a solid solution state in the α-Mg or as a second phase. The role of the Al element has been addressed, and it has been shown that the anti-corrosion ability of Mg alloys is clearly improved when the Al content reaches 10 wt% [20]. In both the (Mg–Al–Mn) AM and (Mg–Al–Zn) AZ series of alloys, β-Mg_17_Al_12_ and Al–Mn phases are the most common second phases. Of these phases, the quantity, morphology, and distribution of the intermetallic Mg_17_Al_12_ have significant effects on the anti-corrosion performance of Mg alloys. A fine β-Mg_17_Al_12_ phase is continuously distributed in the microstructure, which is beneficial to the anti-corrosion performance [21,22]. However, a β-Mg_17_Al_12_ phase with a discontinuous distribution can act as a cathode coupled with the Mg matrix, which will accelerate the corrosion [13,14]. The Mn element exists in the alloy in the form of an Al–Mn phase because of its lower solubility. Under atmospheric corrosion conditions, the anti-corrosion performance is not obviously affected by the Al–Mn phase [15]. However, it acts as a cathode in corrosive solutions [23].

There is a common phase, Mg–Al eutectic, which, although its content is low, also has a vital influence on the anti-corrosion behavior of the AZ and AM series of alloys. For example, some investigations showed that an Mg alloy with an Al content of 8.0 wt% (AZ80 Mg alloy) has better mechanical properties [24] and corrosion resistance among the AZ series, even having better corrosion resistance than AZ91D alloy [25,26,27]. Many eutectics act as good corrosion-inhibitive microstructures, which are responsible for the high anti-corrosion performance of AZ80 [26,27]. The AZ80 alloy is composed of three phases: α-Mg, Al–Mn, and Mg_17_Al_12_. Thus, the Al–Mn phase may take some effect on the corrosion behavior of the AZ80 alloy. Some investigations have reported the corrosion behavior of the respective single phases β-Mg_17_Al_12_, α-Mg, and Al_8_Mn_5_. However, the role of the Mg–Al eutectic in Mg alloys have not been understood deeply. Moreover, AZ80 alloy has relatively good anti-corrosion performance because of the existing eutectics. An investigation by Wei et al. [28] showed that the eutectic has better performance in anti-corrosion than the α-Mg phase in AM60 alloy in a polluted atmospheric environment. A similar result was obtained by Shoesmith et al. [3] in a study of the AM50 alloy in a solution containing chloride ions. For the reasons stated above, the Mg–Al alloy was fabricated in this investigation, consisting of α-Mg dendrites and Mg–Al eutectics. No Mn element was added to the Mg–Al alloy, thus avoiding any influence from an Al–Mn phase. It is very useful to clarify in more detail the corrosion process of the Mg–Al alloy in acid aqueous solutions. Some investigations show that researchers used H_2_SO_4_ solution as the de-alloying electrolyte for Mg-containing alloys. For example, in investigations by Li et al. [29] and Bian et al. [30], nanoporous copper and silver were prepared using Ag–Mg–Ce and Mg–Cu–Y alloys as raw materials, respectively. Aqueous sulphuric acids (H_2_SO_4_) in 0.04 and 0.1 mol/L concentrations were respectively applied as de-alloying solutions to dissolve the α-Mg phase. Thus, choosing the H_2_SO_4_ solution as the corrosive solution, on one hand, will mean the corrosion behavior is clarified during the immersion test. Moreover, whether the H_2_SO_4_ solution is a proper selective corrosive agent for Mg–Al alloys or not can be known at the same time. Many investigations also showed that the Mg–Al alloys have nearly the same corrosion behavior in different strong acid aqueous solutions. But the corrosion behavior in weak acid solutions, for example, acetic acid (HAc), for the Mg–Al alloys has rarely been reported up to now. For these reasons, HAc was selected as a corrosive solution in the investigation so that some novel experimental phenomena might also be present.

## 2. Experimental

### 2.1. Materials

In the experiment, pure Mg and commercial pure Al were used. The Mg–Al alloy was melted at 720 °C in a furnace. The Al weight content is 32.5%. During the melting, the protection gas Ar was used. Finally, the melt was cast into a mould. When the ingot cooled to room temperature, the Mg–Al alloy was obtained. The fabrication process of the single-phase Mg_17_Al_12_ (SP Mg_17_Al_12_) was the same with that of the Mg–Al alloy. In the experiment, the corrosion potential difference between α-Mg and Mg_17_Al_12_ phase of Mg–Al alloy is difficult to test. However, this difference is an important parameter for de-alloying process. Thus, the solid solution AZ91D (SS AZ91) and AZ61 (SS AZ61) alloys were also used in the experiment. The chemical composition of AZ91D and AZ61 alloys are shown in Table 1. The AZ61 alloy was subjected to solid solution at 400 °C for 12 h and then quenched in water at 30 °C. The AZ91D alloy was treated at 420 °C for 24 h and then quenched in water at 30 °C. SP Mg_17_Al_12_ was fabricated, which represents the cathodes of the eutectic. Pure Mg, SS AZ61, and SS AZ91D were used to act in the role of anode.

### 2.2. Corrosion Medium

Acetic acid (analytical reagent, mass fraction: 99.9 wt%) was used to prepare the HAc aqueous solutions. First, 19 mL of 99.9 wt% HAc was transferred into a 1000 mL beaker and diluted to 1000 mL with deionized water. Five parallel HAc solutions were prepared and stirred for 30 min using an electromagnetic stirrer. The pH values were tested using an electronic pH tester. The pH values of the solutions were in the range 1.00–1.10, which illustrates that the solution preparation method can keep the solution pH stable.

In the present study, 0.05 mol/L H_2_SO_4_ aqueous solutions was chosen as a possible corrosive medium for de-alloying treatment of an active Mg–Al alloy. The corrosion behavior of the alloy in H_2_SO_4_ solution was also investigated. The corrosive solution was prepared in the following steps. The corrosive medium was obtained by adding 3 mL of 98.3 wt% concentrated H_2_SO_4_ to 1000 mL of deionized water. The pH of the solution was stable in the range 1.00–1.05.

### 2.3. Weight Loss

The dimensions of specimens were 15 mm × 15 mm × 2 mm. Specimens were ground with sand paper and rinsed with acetone and deionized water. Specimens were weighed respectively using a Mettler Toledo ML204 electronic balance with a resolution of 0.1 mg; original weight is represented by *m*_0_. After being corroded for various times in the acid solution and removing the corrosion products by chromic (20 wt% CrO_3_ + 1 wt% AgNO_3_), the specimens were weighed again, and the value was recorded as *m*_1_. Five replicas of the Mg–Al alloy were immersed in the corrosive medium for 30, 60, and 90 min. Every specimen was immersed in a beaker with 300 mL acid solution. The following formula was used to calculate the corrosion rate:

(1) $$\nu_{m}=\frac{m_{1}-m_{0}}{St}\;(g\cdot {\mathrm{cm}}^{-2}\cdot h)$$

where *v*_m_ is the corrosion rate, *t* the corrosion time, and *S* the specimen’s surface area.

### 2.4. Hydrogen Evolution

The average corrosion rates can be assessed by hydrogen evolution [31]. In this investigation, the hydrogen evolution volumes of specimens in H_2_SO_4_ solution were recorded once every minute because of the low pH of the corrosive medium. The hydrogen evolution rate of the specimen in HAc solution was lower, and the total hydrogen volume was recorded once every 2 min. The corrosion rate was obtained from the following formula:

(2) $$\nu_{H}=\frac{v_{1}-v_{0}}{St}\;\left(\mathrm{mL}\cdot {\mathrm{cm}}^{-2}\cdot h\right).$$

### 2.5. pH Measurement

The acid solution pH was measured by a precision pH meter (Mettler Toledo SG2, ±0.01, Zurich, Switzerland) during the immersion experiment. By measuring the solution pH, the H^+^ concentration can be obtained immediately. The solution pH greatly affects the corrosion behavior of Mg-based materials [32,33,34]. If the solution pH remained unchanged after the experiment had finished, the real corrosion behavior of the specimen in the acid solution given could be determined.

### 2.6. Electrochemical Measurements

The electrochemical corrosion behavior of a sample was characterized by using a PGSTAT30 electrochemical workstation. The samples used for the electrochemical test were similar to those for the immersion test. The F029 corrosion test system was used to measure the polarization curves. Each test was conducted by using 300 mL acid solution coupled with three-electrode system. Polarization measurements were carried out in a corrosion cell containing 300 mL corrosive acid solution using a standard three-electrode system. The saturated KCl calomel electrode (SCE) served as the reference electrode. The sample was encased in resin, with 1 cm^2^ being left exposed as the working electrode. The counter-electrode was a platinum sheet. The electrochemical test was carried out in the potential range of −2.0 to 0 V with a rate of 0.5 mV/s. Each test was conducted by using three parallel specimens. The electrochemical parameters were gained by using the Tafel extrapolation method. 

### 2.7. Surface Analysis

The corroded specimen surface was examined by a scanning electron microscope (SEM, Tescan VEGA3, Brno, Czech Republic) attached with an energy-dispersive spectrometer (EDS). Combining the line-scanning and area-scanning methods, the chemical composition on the sample surface can be analyzed effectively.

## 3. Experimental Results

### 3.1. Microstructural Characterization

**Figure 1:**
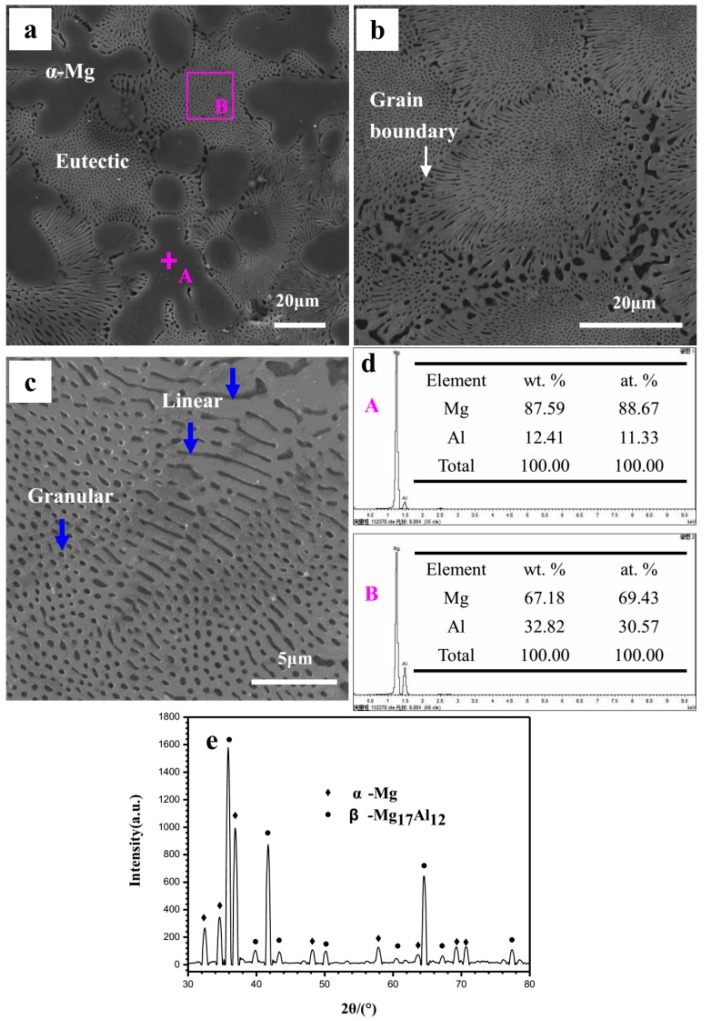
The cast Mg–Al alloy’s microstructure: (**a**) Cast Mg–Al alloy; (**b**) SEM image of eutectic structure at high resolution; (**c**) linear and granular like α-Mg phases in eutectic; (**d**) EDS analysis results at sites A and B in the SEM image (**a**); (**e**) the Mg–Al alloy’s XRD test result.

The Mg–Al alloy is composed of α-Mg matrix and eutectic, as shown in Figure 1a. The α-Mg phase shows large dendrites and is randomly distributed in the matrix. By contrast, the eutectic is very fine. Figure 1b is an enlarged view of the eutectic microstructure. It can be seen that the eutectic is about 30 μm in grain size. The eutectic near to the grain boundary has a radial distribution. In the eutectics, the α-Mg phase exhibits two kinds of morphologies: Typically, round-like with a radius of 0.5 μm and bar-like with a width of 0.5 μm (Figure 1c). It should be noted in particular that the round-like α-Mg phase may possibly be an outcrop on the specimen surface of the bar-like phase. The presence of two morphologies in the eutectic is due to different α-Mg phase characters on the specimen surface: Point-like eutectic and feather-like eutectic. The α-Mg phase in the alloy is easily corroded, and its distribution has a significant influence on the corrosion behavior of specimen. The real morphology of the α-Mg phase in the eutectic may be revealed during the corrosion process, as discussed in Section 4. In this investigation, the Mg–Al alloy has a high Al content, and thus the Al content of α-Mg dendrites formed in its microstructure may be different from that in the common alloys AZ31, AZ91, AM60, etc. The EDS results in Figure 1d show that the Al content of the α-Mg dendrites is 12.4 wt% (the average value of seven test points, including the point A), which is higher than that of the common Mg alloys [35] and will inevitably affect the specimen’s corrosion behavior. The eutectic in site B has an Al content of 32.8 wt%, which is quite close to the ideal value, 32.3 wt% [36]. The XRD pattern shows that the Mg–Al alloy only consists of α-Mg and Mg_17_Al_12_ phases (Figure 1e), which agree with the results shown in Figure 1a.

### 3.2. Polarization Curves

**Figure 2:**
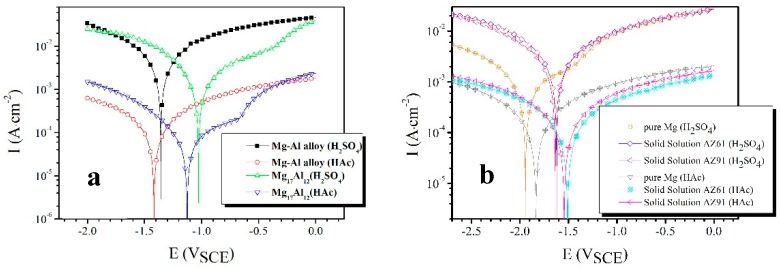
Polarization curves of (**a**) the Mg–Al alloy and SP Mg_17_Al_12_ in H_2_SO_4_ and HAc solutions and (**b**) pure Mg, SS AZ61, and SS AZ91 in H_2_SO_4_ and HAc solutions, respectively.

The polarization curves results are shown in Figure 2. It isclear that the corrosion potential (*E*_corr_) of SP Mg_17_Al_12_ is more positive than that of the Mg–Al alloy in H_2_SO_4_ solution, and the same results are obtained in HAc solution. GPES software in combination with the electrochemical workstation was used to measure the electrochemical parameters of the specimens, which are shown in Table 2. *i*_corr_ is the corrosion current density. The cathodic branches and *E*_corr_ are used to fit the *i*_corr_ [37,38].

The SP Mg_17_Al_12_ has a 330 mV more positive corrosion potential than the Mg–Al alloy in H_2_SO_4_ solution (Figure 2a). The similar results were observed in HAc solution, and the potential difference is 289 mV. The *i*_corr_ can be used to evaluate the anti-corrosion ability of an alloy [39]. The *i*_corr_ values of the Mg_17_Al_12_ in H_2_SO_4_ and HAc solution are both about half that of the Mg–Al alloy. Apparently, the SP Mg_17_Al_12_ has higher anti-corrosion performance than the Mg–Al alloy.

The corrosion potential difference (α-Mg phase and Mg_17_Al_12_) is difficult to measure in an acid solution, and so we fabricated several alloys, such as the SP Mg_17_Al_12_, pure Mg, solid solution AZ61, and AZ91D. The SP Mg_17_Al_12_ could be regarded as the cathode when the eutectic alloy was immersed in solution. Pure Mg, SS AZ61, and SS AZ91 were considered as the anode and their polarization curves were tested (Figure 2b) to determine the influence of Al content on corrosion resistance. From the polarization curves and their electrochemical parameters, one can find that all selected candidate alloys for the anode above have nearly the same order in the corrosion current density in H_2_SO_4_ solution and HAc solution, respectively. But an obvious difference appears in the corrosion potential. Because the pure Mg has a very low corrosion potential compared with the SS AZ61 and the SS AZ91, it is unsuitable to represent the anode of eutectics. There is a little difference between the SS AZ61 and the SS AZ91 in the corrosion potential, and the latter has a higher Al content. For this reason, the SS AZ91 was selected as a replacer of the anode of the eutectic in this investigation. The measured corrosion potential difference between the SS AZ91 and the SP Mg_17_Al_12_ is 610 mV in H_2_SO_4_ solution and 418 mV in the HAc solution, respectively.

### 3.3. Corrosion Rate

**Figure 3:**
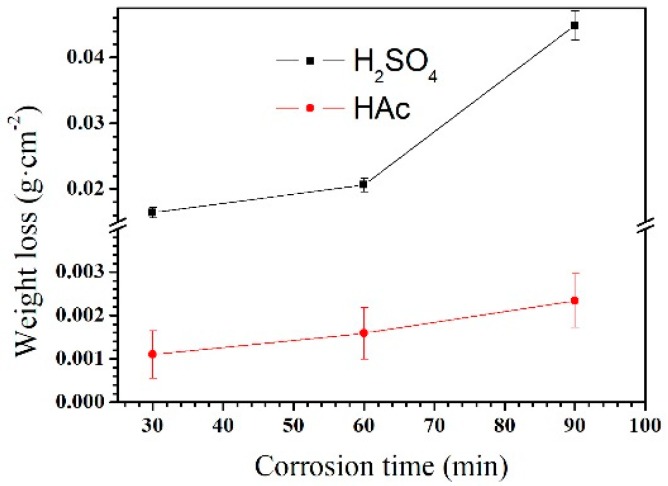
Weight losses of the Mg–Al alloy in H_2_SO_4_ and HAc solutions for 30, 60, and 90 min.

**Figure 4:**
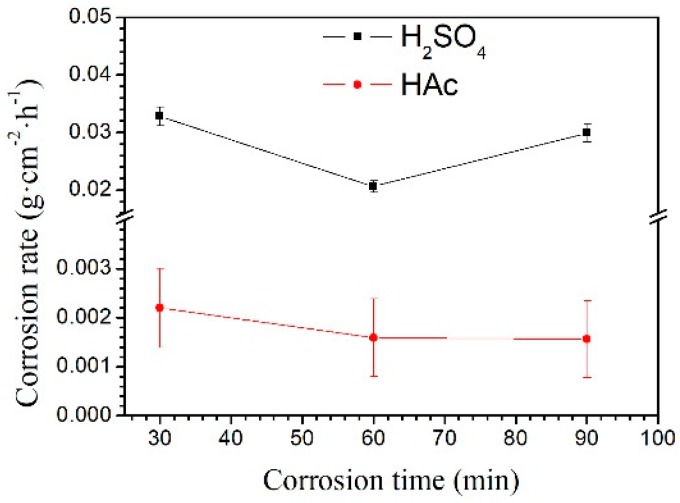
The Mg–Al alloy’s corrosion rates in H_2_SO_4_ and HAc solutions for 30, 60, and 90 min.

The weight loss rate of the Mg–Al alloy in H_2_SO_4_ solution is shown in Figure 3. The weight loss rate is not linear with the corrosion time. Within the corrosion time range of 30–60 min, the weight loss rate increases slowly. However, it increases rapidly in the range 60–90 min. When specimens were immersed in HAc solution, the weight loss rate is linear to the corrosion time. In the experiment, the weight loss rate reflects the weight change per unit area; by contrast, the corrosion rate, shown in Figure 4, is a better reflection of the corrosion speed for a specimen. In the first 30 min, specimens had a higher corrosion rate than that after 60 min immersion in H_2_SO_4_ solution. However, with the prolong of corrosion time to 90 min, the corrosion rate increased again. In HAc solution, the specimens exhibit a steady corrosion rate.

**Figure 5:**
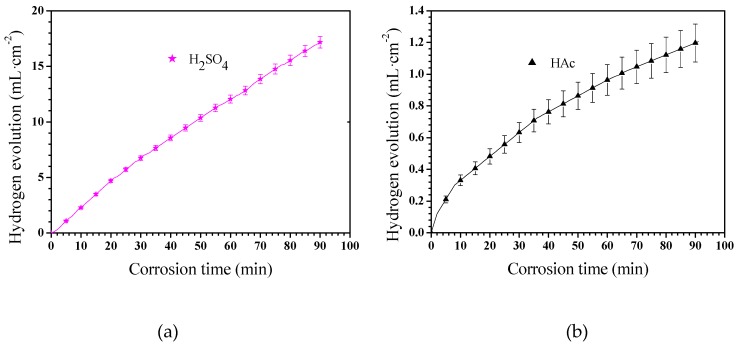
The Mg–Al alloy’s hydrogen evolutions in H_2_SO_4_ (**a**)and HAc (**b**)solutions for 90 min.

The corrosion rate of Mg–Al alloys can also be assessed by hydrogen evolution measurement [40]. The Mg–Al alloy’s hydrogen evolution results in these two solutions is shown in Figure 5. The Mg–Al alloy’s hydrogen evolution volume per unit area in H_2_SO_4_ solution shows an approximately linear dependence on the corrosion time from 0 to 90 min (Figure 5a). The hydrogen evolution volume of the specimen in HAc solution slowed down gradually with extended corrosion time, which indicates that the corrosion process was suppressed (Figure 5b). Specifically, the hydrogen evolution rate was rapid in the first 8 min, after which the hydrogen evolution process progressed sluggishly.

### 3.4. Corrosion Morphology

**Figure 6:**
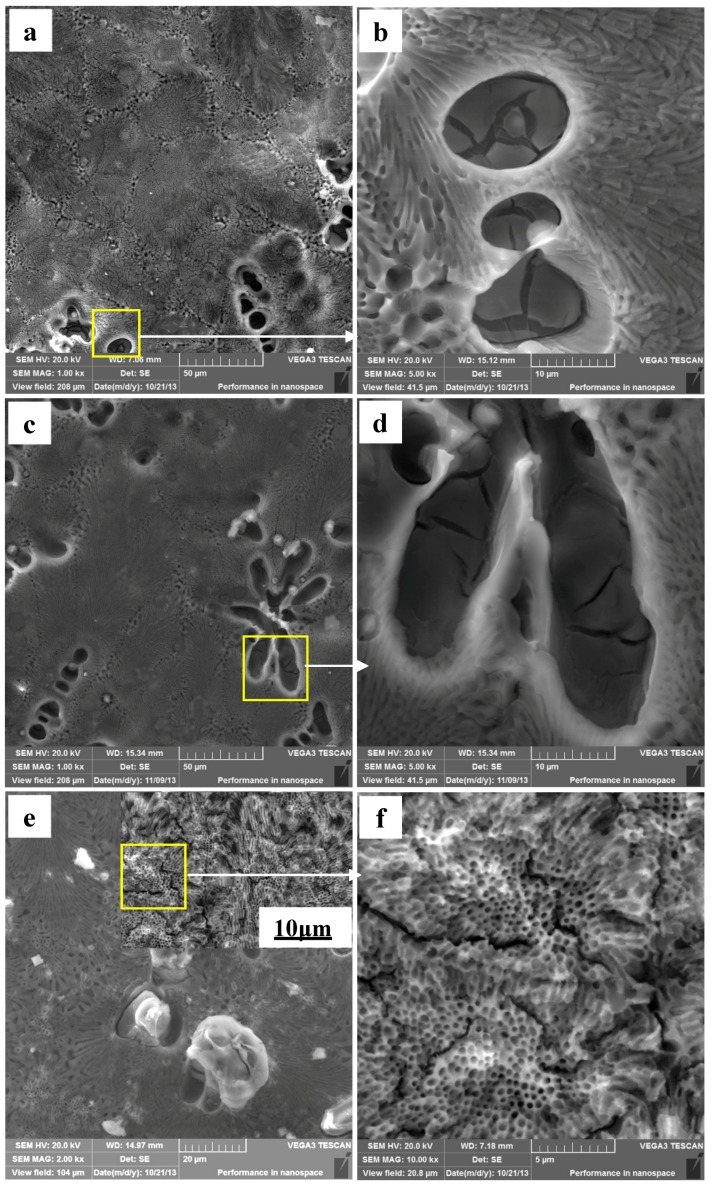
The corroded Mg–Al alloyspecimen’s corrosion morphologies gained from H_2_SO_4_ solution for different immersion times: (**a**,**b**) 30 min; (**c**,**d**) 60 min; (**e**,**f**) 90 min.

The Mg–Al alloy’s surface morphologies after immersion for various times in H_2_SO_4_ solution are shown in Figure 6. The Mg–Al specimen’s corroded surface morphology after immersion for 30 min is shown in Figure 6a. Overall, the corroded specimen surface is uniform, and the α-Mg dendrites are corroded more severely than the eutectics. Figure 6b is an enlarged detailed view of the corroded α-Mg dendrite in Figure 6a and shows that some deeper pits formed at the α-Mg dendrite areas and that these α-Mg phases are not corroded completely, with some corrosion films and cracks being formed on it. It indicates that the protective effect of these films is not enough to hinder the corrosion of the matrix. Some corrosion products are observed at the border of the α-Mg dendrites for specimens immersed for 60 min (Figure 6c). In Figure 6d, it can be seen that discontinuous films are present on the α-Mg dendrite surfaces. The corrosion products on the specimen surfaces increase in quantity gradually as the immersion time is prolonged to 90 min (Figure 6e). Under higher magnification, Figure 6f shows that the eutectic surface becomes uneven, with the appearance of many cracks. All these results illustrate that the specimens are severely corroded. In general, the eutectics have better corrosion resistance than α-Mg dendrites, no matter how long the specimen is immersed in H_2_SO_4_ solution.

**Figure 7:**
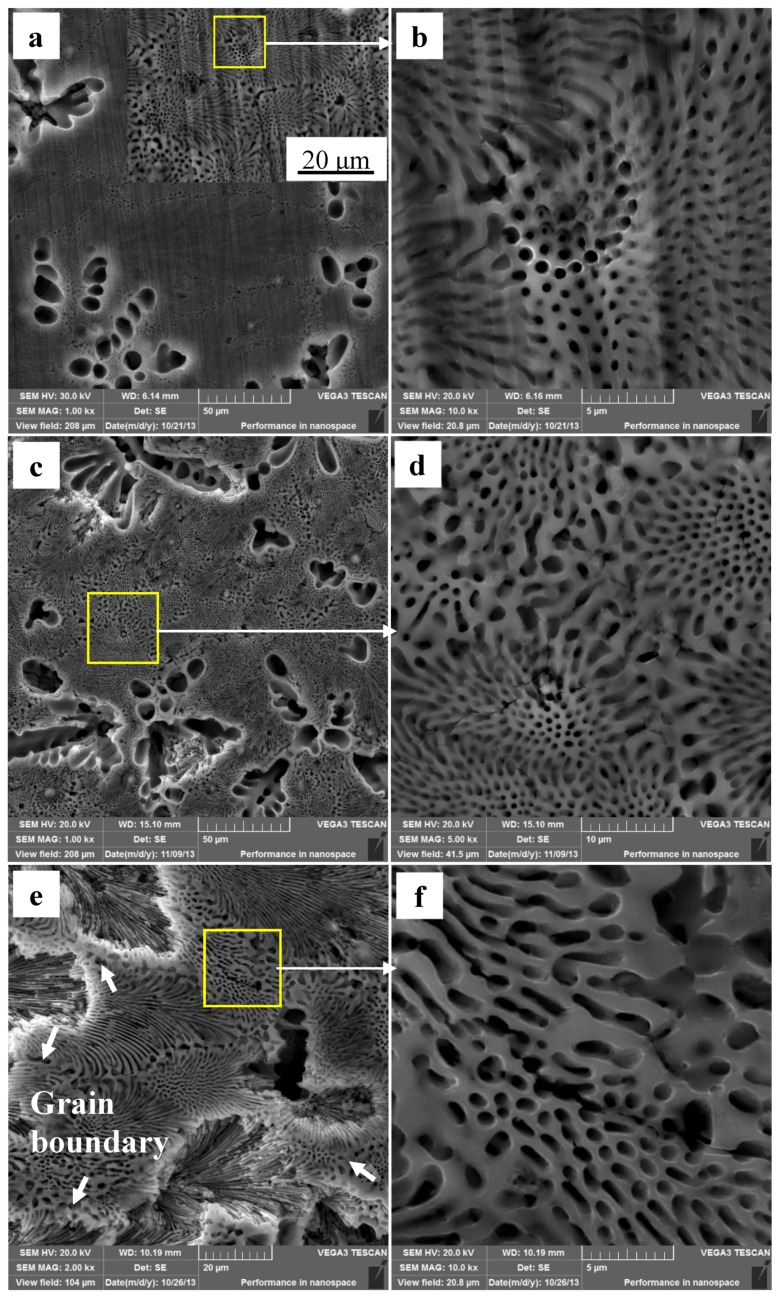
The corroded Mg–Al alloyspecimen’s corrosion morphologies gained from HAc solution for different immersion times: (**a**,**b**) 30 min; (**c**,**d**) 60 min; (**e**,**f**) 90 min.

Figure 7 shows the Mg–Al alloy’s surface morphologies after immersion in HAc solution. It is rather obvious that the corrosion behavior of the Mg–Al specimen in these two acid solutions is very different. The α-Mg dendrites are corroded completely, and the bottoms and borders of the pits are rather smooth after the specimen has been immersed for 30 min (Figure 7a). However, only part of the α-Mg phase of eutectic disappears (Figure 7b). Figure 7c shows the corrosion morphology of a specimen immersed for 60 min. It is found that the eutectic regions of the alloy surface are still smooth, as was the inner wall of the pit at the site of the original α-Mg dendrite (Figure 7d). The enlarged local image in Figure 7c shows that the α-Mg phase in the eutectic is almost entirely corroded (Figure 7d). Some feather-like eutectics peel off from the matrix when the corrosion time is prolonged to 90 min (Figure 7e), which results in the formation of corrosion pits. The borders of these pits are very near to the grain boundary, which is the shallowest corrosion site for a fixed pit. Moreover, the pit center has the deepest corrosion depth. By comparison, the point-like eutectics have not peeled off from the matrix, although a few cracks have formed in them (Figure 7f). 

### 3.5. Cross-Sectional Morphology

**Figure 8:**
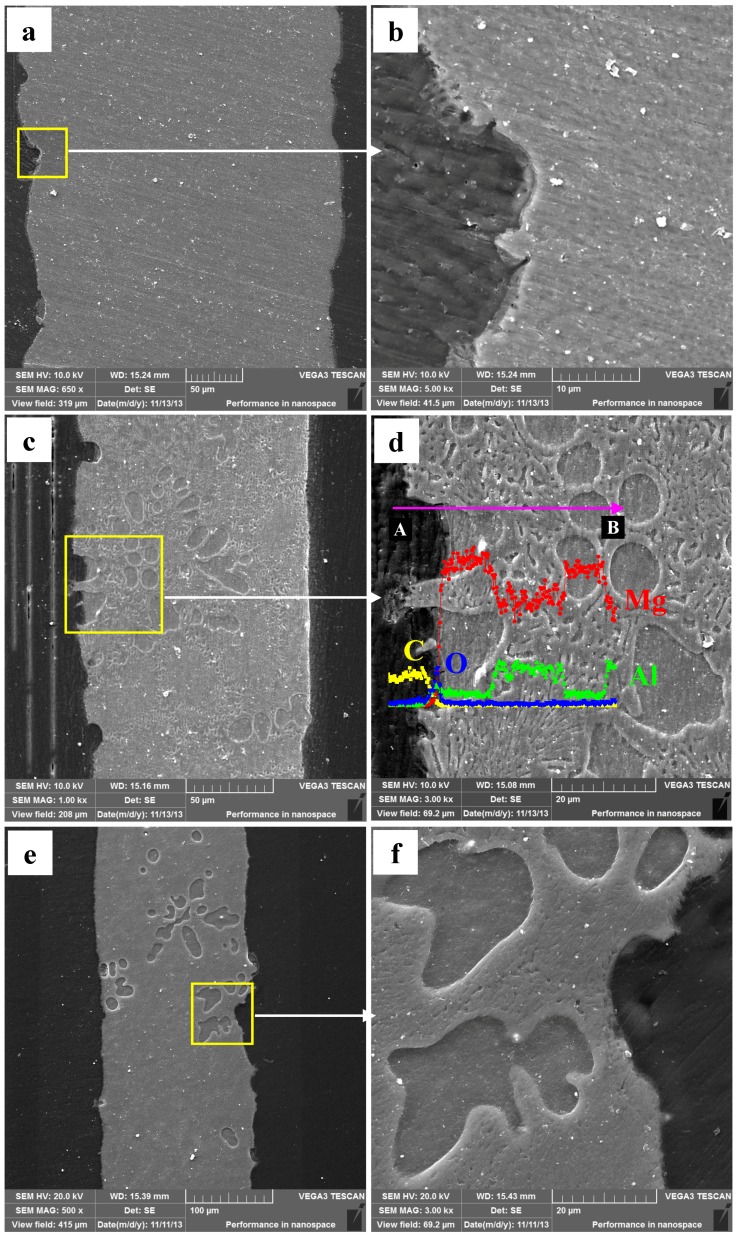
The corroded Mg–Al alloy specimen’s cross-sectional morphologies obtained from H_2_SO_4_ solution for different immersion times: (**a**,**b**) 30 min; (**c**,**d**) 60 min; (**e**,**f**) 90 min.

The Mg–Al alloy’s cross-sectional morphologies are gained after immersion for various times in H_2_SO_4_ solution (Figure 8). On the specimen surface, there are some randomly distributed deep pits instead of a smooth surface (Figure 8a). The α-Mg phases in these pits are not corroded completely. On the uncorroded α-Mg dendrite surface, an incomplete film is formed during the immersion test (Figure 8b), the same results shown in Figure 6b. A porous layer about 3 μm thick is left in some areas after the immersion test. After corrosion for 60 min, a similar specimen surface morphology is observed (Figure 8c), and the character of the α-Mg dendrite corrosion also resembles this (Figure 8b). The EDS results illustrate the corrosion films formed had high Al and O contents, which might imply that these films are mainly composed of Al-rich oxides (Figure 8d). Moreover, a porous layer may be also present, with a thickness of 3 μm. After immersing for 90 min, the specimens’ cross-sectional morphologies are obtained and shown in Figure 8e,f. No porous layer is observed, which may be related to the periodic peeling off of these layers during immersion.

**Figure 9:**
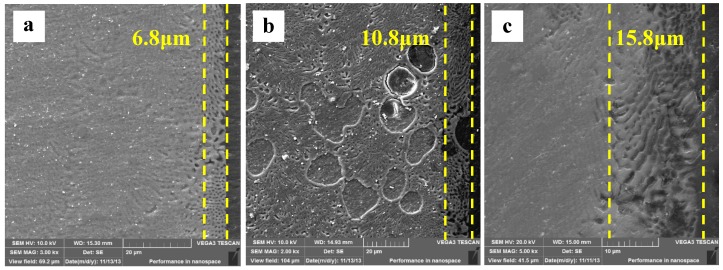
The corroded Mg–Al alloy specimen’s cross-sectional morphologies gained from HAc solution after immersion for different times: (**a**) 30 min, (**b**) 60 min, (**c**) 90 min.

After immersing specimens in HAc solution for 30 min, the cross-sectional morphologies are characterized (Figure 9a). There are obvious differences in cross-sectional morphology between Figure 9a and Figure 8. First, the specimen surface remains smooth, is slightly corroded, and is not like the uneven one shown in Figure 9a. Second, the enlarged detail shows that the α-Mg dendrites as well as the α-Mg phase of the eutectics are both corroded, with only the Mg_17_Al_12_ framework left. The results show that the corroded depth, measured by line scanning, is extended about 6.8 μm to the deeper matrix. The Mg content in the corroded layer increases from the surface to the interior of the specimen, which indicates that the α-Mg phase in the eutectics is not corroded completely throughout the layer. In other words, the degree of de-alloying is not complete in the 6.8 μm thick layer. With the immersion time prolonged to 60 min, the specimen surface is still smooth. The corroded depth is increased to 10.8 μm (Figure 9b). Some deeper pits form at the original α-Mg dendrite sites owing to their poor corrosion resistance. The maximum depth of these pits reaches 40 μm, which can be demonstrated by the resin in these pits. With the immersion time increased to 90 min, the corrosion depth is up to 15.8 μm (Figure 9c), approximately 10 μm of which is a completely de-alloyed layer. Overall, the corroded depth at the sites of the α-Mg dendrites is deeper than that in the eutectic regions. During immersion testing, the original Mg_17_Al_12_ framework is preserved, which suggests that the Mg–Al alloy is corroded in a selective form in HAc solution.

### 3.6. Corrosion Behavior of SP Mg_17_Al_12_ and Mg–Al Alloy

**Figure 10:**
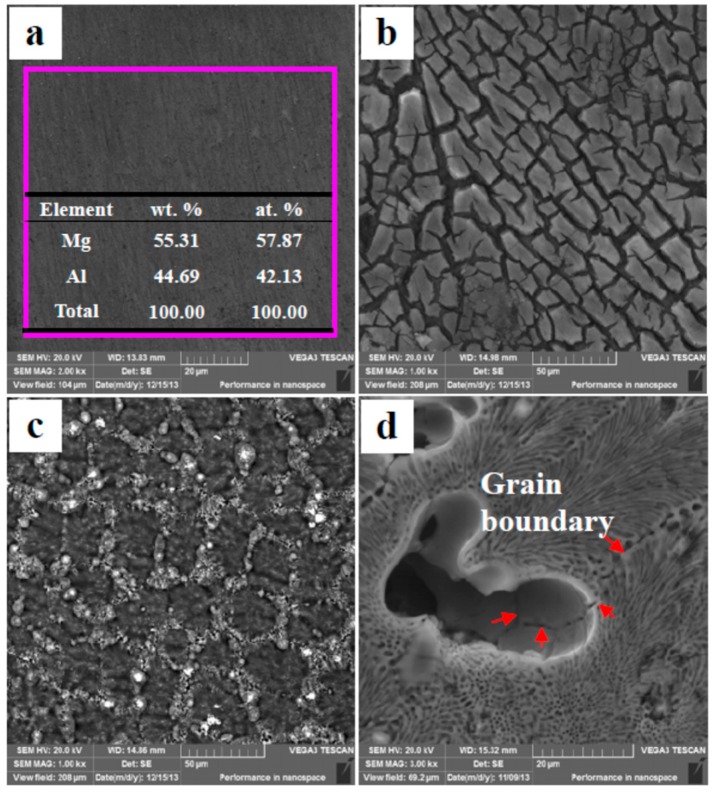
Surface morphologies of the SP Mg_17_Al_12_ and the Mg–Al alloy: (**a**) Original morphology of the SP Mg_17_Al_12_ (un-etched); (**b**) the SP Mg_17_Al_12_ alloy’s corrosion morphology after 90 min of immersion in H_2_SO_4_ solution; (**c**) the SP Mg_17_Al_12_ alloy’s corrosion morphology after 90 min of immersion in HAc solution; (**d**) the Mg–Al alloy’s corrosion morphology after 30 min of immersion in HAc solution.

There are two phases in the Mg–Al alloy, α-Mg and Mg_17_Al_12_. The β-Mg_17_Al_12_ is a cathode phase in microstructure, so the corrosion behavior of the whole specimen is significant affected by the α-Mg. The original morphology of cast Mg_17_Al_12_ is shown in Figure 10a. The EDS results are consistent with the ideal composition of the Mg_17_Al_12_. After immersion for 90 min in H_2_SO_4_ solution, many cracks occur on the Mg_17_Al_12_ specimen surface, and some fragments detach from the matrix (Figure 10b), which illustrates that the SP Mg_17_Al_12_ do not have sufficient resistance to corrosion in H_2_SO_4_ solution. Despite that the β-Mg_17_Al_12_ phase has a relative high Al content and an Al-rich film forms on the specimen surface, these advantages cannot hinder corrosion. Al_2_O_3_ is an amphoteric oxide and is not stable in H_2_SO_4_ solution. When the Mg–Al alloy is immersed in H_2_SO_4_ solution, Al-rich films also form on the α-Mg dendrite surfaces (Figure 8d). As a result, a corrosion attack occurs at nearly the same time over the whole specimen surface (Figure 8), although the two phases in the alloy show a great difference in corrosion potential. The corrosion occurs in the α-Mg dendrites only a little earlier than that in the β-Mg_17_Al_12_ phases (Figure 8c,d).

The corrosion morphologies of the SP Mg_17_Al_12_ inHAc solution are shown in Figure 10c. No fragments peel off from the matrix, which illustrates that the Mg_17_Al_12_ has higher anti-corrosion ability in HAc solution. Meanwhile, the large corrosion potential difference between two phases leads to the β-Mg_17_Al_12_ phase acting as an effective cathode in a corrosive environment. Thus, the α-Mg phase is corroded first in the HAc solution. In the Mg–Al alloy, the eutectics exhibit better corrosion resistance than the α-Mg dendrites, which will be corroded preferentially (Figure 7). The Mg–Al alloy’s corrosion morphology is obtained from HAc solution after immersion for 30 min and is shown in Figure 10d. The grain boundary can be seen clearly, which indicates that the α-Mg dendrites are eaten away completely, although the α-Mg phase in the eutectic is not, as also is shown in Figure 7b. In the eutectic microstructures, the network β-Mg_17_Al_12_ phase mainly plays a positive role in corrosion resistance, similar to the experiment results gained by using die-cast AZ91 Mg alloy [41,42]. Thus, the eutectics have higher ability of anti-corrosion than the α-Mg dendrites. The results also show the α-Mg phase in the eutectics is corroded more slowly.

The data in Table 2 show that Mg_17_Al_12_ and SS AZ91D alloys have nearly the same value in *i*_corr_ when they were immersed in H_2_SO_4_ solution. However, Mg_17_Al_12_ has a larger value than SS AZ91D alloy in HAc solution. The results above indicate that Mg_17_Al_12_ and SS AZ91D alloys have nearly the same corrosion resistance in H_2_SO_4_ solution. By contrast, Mg_17_Al_12_ has higher anti-corrosion ability than SS AZ91D in HAc solution. Therefore, the selective corrosion can occur in HAc solution, but cannot occur in H_2_SO_4_ solution for Mg–Al alloy (Figure 7 and Figure 8).

## 4. Discussion

### 4.1. Corrosion Characters Analysis

The Mg–Al alloy’s average corrosion rate in H_2_SO_4_ solution for 30 min is higher than that of the alloy immersed for 60 or 90 min (Figure 4). Of these specimens, the one that is corroded for 60 min has the lowest average corrosion rate. The weight loss data (Figure 3) show that the weight loss of the specimens is 44.9 mg·cm^−2^ after corrosion for 90 min. During this corrosion process, each corrosion period contributes 36.6%, 9.4%, and 54% of the total weight loss in the time ranges 0–30 min, 30–60 min, and 60–90 min, respectively. According to results, Mg–Al alloy’s hydrogen evolution volume is 17.2 mL·cm^−2^ in H_2_SO_4_ solution for 90 min (Figure 5). The hydrogen evolution volume for the respective 30 min corrosion time periods account for 39.3%, 30.8%, and 29.9% of the total value. At the beginning of the 30 min, the hydrogen evolution volume and weight loss per unit area account for nearly the same proportion of their corresponding total values for 90 min. When the corrosion time is longer than 30 min, the proportion of hydrogen evolution relative to the total value for a specimen immersed for 90 min in each time range, 30–60 min and 60–90 min, is obviously different from the relative proportion of weight loss, because of the periodic peeling off from the sample surface layer in the H_2_SO_4_ solution. Before the surface layer peels off, the hydrogen evolution volume of the samples increased continuously, with a lower weight loss. When the surface layer peels off, the weight loss increases suddenly. However, the fragment is also corroded, producing hydrogen. Thus, the hydrogen evolution volume increases steadily, although the weight loss shows an obvious fluctuation.

The changes in corrosion rate are different in these two solutions. The weight loss of the specimen is 2.3 mg·cm^−2^ after immersion for 90 min. During the immersion testing, for the three time ranges 0–30 min, 30–60 min, and 60–90 min, the weight loss account for 47%, 21%, and 32%, respectively, of the total value, and the hydrogen evolution volume account for 52%, 27%, and 21%, respectively, of the total value. The cross-sectional morphologies shown in Figure 9a–c indicate that the specimen is corroded in a selective form in HAc solution. The α-Mg phase is corroded preferentially, leaving a porous Mg_17_Al_12_ framework. The EDS results show the total thickness of the corroded layer is about 15.8 μm after immersion for 90 min. The thicknesses of the corroded layer are 6.8 and 10.8 μm after immersion for 30 and 60 min, respectively. Thus, the corrosion depth in each time range accounts for 43%, 25%, and 32% of the total value, which is basically consistent with the ways in which the weight loss and hydrogen evolution volume change. After immersion for 30 min in HAc solution, the specimen’s corrosion rate slows down, which may be due to the following reasons. First, the H^+^ concentration at the corrosion interface decreases as the corrosion proceeds. Second, the contact area is reduced between the corrosive solution and the α-Mg phase, which has a poor corrosion resistance. The porous surface layer hinders the corrosive ion exchange. The α-Mg dendrites are corroded rapidly and completely in the first 30 min. All of these factors result in a slower corrosion rate in the following corrosion process.

### 4.2. Grain Orientation’s Role in Corrosion Process 

In this section, ‘grain orientation’ means the growth direction of α-Mg and β-Mg_17_Al_12_ in eutectic.

Figure 8 shows the corrosion rate of α-Mg dendrites is higher than that of the eutectic microstructures. The eutectics peel off in 3 μm thicknesses from the specimen surface to its center. During the immersion test, Al-rich films would be formed on the α-Mg dendrite surface, which was also found by Pardo et al. [26,27]. However, this film is not sufficiently dense (Figure 6b,d) and cannot effectively prevent corrosion. Even if the film is dense enough, it will not be able to inhibit corrosion yet, because of the amphoteric nature of the oxide Al_2_O_3_, which is unstable in dilute H_2_SO_4_ solution. The Al-rich films can easily be observed as a result of the α-Mg phase being corroded preferentially, with the remaining Al atoms being deposited from solution on the α-Mg phase surface. This process may be similar to the re-deposition of atoms during de-alloying [43,44]. In this study, the Al content of the α-Mg dendrites is nearly as high as the Al atom’s solid-solution limit in the Mg matrix, which may lead to Al atoms being readily deposited on the alloy surface and then corroded to form oxide. During the corrosion test, cracks formed in the β-Mg_17_Al_12_ phase of the eutectic. Despite the good corrosion resistance of the eutectic, cracks may result in a layer peeling off from the specimen surface. In summary, the Mg–Al alloy’s α-Mg dendrites are corroded preferentially in H_2_SO_4_ solution, followed by the eutectic. The macro-morphology shows the general corrosion and the approximately uniform thinning of the sample thickness. The grain orientation has little effect on Mg–Al alloy’s corrosion behavior in H_2_SO_4_ solution.

There is a significant difference in corrosion behavior in two solutions. Specifically, the α-Mg phase is corroded, but the β-Mg_17_Al_12_ phase is hardly affected. The Mg–Al alloy is corroded selectively in HAc solution. The cross-sectional morphology in Figure 9 shows that the specimen thickness remains nearly unchanged after the immersion test. Moreover, the β-Mg_17_Al_12_ phase is left and only some of the feather-like eutectic peels off from the specimen surface after corrosion for 90 min (Figure 7). These two results are the best evidence of the occurrence of selective corrosion.

From the original microstructure, it can be seen that the diameter or width of the α-Mg phase of the eutectic is about 0.5 μm and its length is about 5 μm at least, although generally this value will be in the range 10–20 μm. Thus, the α-Mg phase in the eutectic can be considered as consisting of submicron rods instead of submicron balls. The thickness of the de-alloyed layer reaches 15.8 μm, with that of the completely de-alloyed layer being about 10 μm. At this time, some of the feather-like eutectics peel off from the specimen surface, with this phenomenon occurring over nearly the whole grain. This behavior can be explained as follows. The diameter of α-Mg rods is much less than the de-alloyed layer thickness, which is nearly equal to the length of the rods and the grain size in the eutectic areas. In this situation, when the grain orientation is almost the same as the specimen surface (forming feather-like eutectics), and when the α-Mg phase is corroded completely, the Mg_17_Al_12_ phase finally loses support from its surrounding α-Mg phase and peels off from the matrix. Corrosion pits 5–10 μm deep are then formed in the eutectic areas. These corrosion pits occur because the growth orientation of the corresponding eutectic is parallel to the specimen surface, and the presence of a shallow pit can then lead to some discontinuous β phases peeling off. In contrast, when the grain grows along the direction that is normal to the sample surface, the α-Mg will not be corroded completely, because of the lower de-alloyed layer thickness. Thus, the β phases of the point-like eutectics are still well preserved. The point-like eutectic and feather-like eutectic in the specimen surface thus show different corrosion and peel-off behavior due to the differences in their grain orientation.

**Figure 11:**
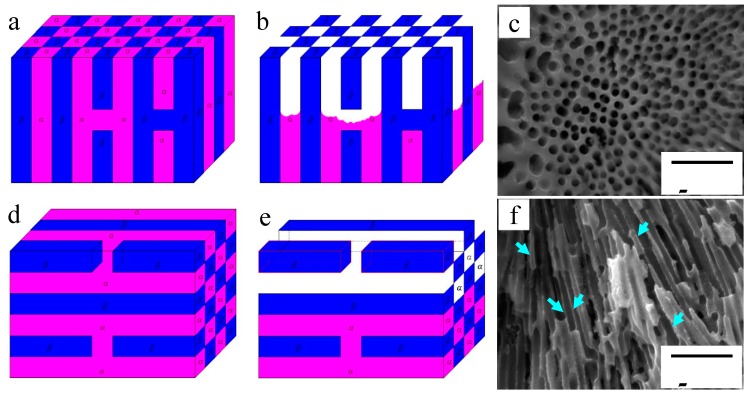
Effect of grain orientation on corrosion mechanism and corrosion morphologies of eutectic microstructures: (**a**,**b**) Schematic diagrams of the point-like eutectic before and after immersion test; (**c**) corrosion morphologies of the point-like eutectic after 90 min of immersion; (**d**,**e**) schematic diagrams of the feather-like eutectic before and after immersion test; (**f**) corrosion morphologies of the feather-like eutectic after 90 min of immersion in HAc solution.

According to the above analysis, these two kinds of eutectics on the specimen surface can be simplified as shown in Figure 11. Schematic diagrams of point-like eutectic before and after corrosion are shown in Figure 11a,b. The porous β-Mg_17_Al_12_ phases are left after immersion for 90 min as shown in Figure 11c, which is the real specimen surface morphology. Figure 11d is a schematic diagram of feather-like eutectic before immersion. The α-Mg near the surface is also corroded because of the presence of discontinuous β-Mg_17_Al_12_ phases. Then, the β-Mg_17_Al_12_ phases in the upper most surface are separated from matrix, with only some sites remaining weakly connect to the matrix (Figure 11e). Although HAc has a smaller corrosive effect on the β phase than on α-Mg, the β phase can be easily detached from the matrix. The real surface morphology of the feather-like eutectic is shown in Figure 11f. After the corrosion test, the β-phase walls are very thin, as indicated by the arrows, which is consistent with Figure 11e. Thus, the immersion tests illustrate the deduction from Figure 1 that the difference in morphologies of the eutectics results from the different grain orientations. In other words, the round-like α-Mg phase of the point-like eutectics is only an outcrop of submicron α-Mg rods on the specimen surface.

### 4.3. Corrosion Behavior Analysis

General corrosion character is observed on Mg–Al alloy’s surface in H_2_SO_4_ solution. Microanalysis results show that the α-Mg dendrites are corroded faster than the β phase, and Al-rich films will form on the surface of the former at the same time. Because of its poor corrosion resistance, the β phase begins to corrode before the α-Mg dendrites are eaten up. The formed porous Mg_17_Al_12_ on the specimen surface will peel off layer by layer, with each layer having a thickness of 3 μm. As a result, the corrosion rates of the specimens are very high in H_2_SO_4_ solution. Investigations by Zhang et al. [45] and Atrens et al. [46] show that MgH_2_ will be formed on the Mg alloy’s surface and intermetallics phase’s surface, with this occurring more easily on the latter, when they are immersed in a corrosive solution [47]. Hydrogenation of Mg alloys or Mg/Al intermetallics can accelerate their powdering [48], which may be the basic reason thatthe Mg–Al alloy peels off very rapidly, starting from specimen surface, in H_2_SO_4_ solution. By contrast, the specimens have a low corrosion rate in HAc solution, and no layered peeling can be observed during the tests. 

In HAc solution, the Mg–Al alloy’s corrosion process can be divided into three steps. In the first step, the α-Mg dendrites are corroded preferentially due to the great corrosion potential difference existing in α-Mg and β-phases, and the better corrosion resistance of the β-phase in HAc solution. In the second step, the corrosion spread to the α-Mg phases of the eutectics. In the third step, with the spread of corrosion, the α-Mg phase in β phasesurrounding is eaten up, and the feather-like eutectic then begins to peel off from the matrix, resulting in the formation of a large corrosion pit. 

In summary, the corrosion potential difference is smaller between α-Mg and β phase in HAc solution than that in H_2_SO_4_ solution (Figure 2). In HAc solution, Figure 10 shows the SP Mg_17_Al_12_ has a higher anti-corrosion ability than it in H_2_SO_4_ solution. Thus, the corrosion potential difference is not the only prerequisite for the occurrence of a selective corrosion. Based on the above analysis, in HAc solution, the selective corrosion characteristic is observed in Mg–Al alloy, which possibly results from the following two reasons. (i) Two-phase (α-Mg and β-phase) corrosion potential difference is great enough. (ii) The noble phase, β-Mg_17_Al_12_ phase, has a good corrosion resistance in the de-alloying electrolyte.

## 5. Conclusions

A Mg–Al alloy was fabricated in this investigation and composed of α-Mg dendrites and eutectics. Immersion tests show that the Mg–Al alloy’s corrosion behavior in H_2_SO_4_ solution is considerably different from that in HAc solution.

(1) The general corrosion character is observed in Mg–Al alloy in H_2_SO_4_ solution with a pH of 1.0. Conversely, the Mg–Al alloy is selectively corroded in HAc solution with a pH of 1.0.
(2) In HAc solution, the Mg–Al alloy’s corrosion behavior is affected by the grain orientation. The point-like eutectics show a better resistance to peeling than the feather-like eutectics. In contrast, the similar corrosion character is not observed in H_2_SO_4_ solution.
(3) In both H_2_SO_4_ and HAc solutions, the eutectics show a greater anti-corrosion ability than the α-Mg dendrites.

## Figures and Tables

**Table 1:** Thechemical composition of AZ91D and AZ61 alloys (wt.%)

Alloy	Al	Zn	Mn	Fe	Si	Cu	Ni	Be	Mg
AZ91D	8.91	0.67	0.21	0.0026	0.0331	0.0047	0.00060	0.0009	Bal.
AZ61	6.67	1.03	0.40	0.0036	0.0210	0.0018	0.00051	0.0007	Bal.

**Table 2:** Electrochemical parameters obtained from polarization curves in H_2_SO_4_ and acetic acid (HAc) solutions.

In H_2_SO_4_ Solution	*E*_corr_ (V/SCE)	*i*_corr_ (μA cm^−2^)	In HAc Solution	*E*_corr_ (V/SCE)	*i*_corr_ (μA cm^−2^)
Mg_17_Al_12_	−1.026 ± 0.010	452 ± 208	Mg_17_Al_12_	−1.126 ± 0.009	15.3 ± 7.1
Mg–Al alloy	−1.356 ± 0.023	755 ± 345	Mg–Al alloy	−1.415 ± 0.013	22.8 ± 10.5
Pure Mg	−1.944 ± 0.019	197 ± 91	Pure Mg	−1.835 ± 0.021	29.4 ± 13.5
SS AZ61	−1.621 ± 0.030	385 ± 173	SS AZ61	−1.509 ± 0.017	20.7 ± 9.4
SS AZ91D	−1.636 ± 0.025	453 ± 159	SS AZ91D	−1.544 ± 0.011	25.0 ± 11.2
